# Immune activation and inflammation in lactating women on combination antiretroviral therapy: role of gut dysfunction and gut microbiota imbalance

**DOI:** 10.3389/fimmu.2023.1280262

**Published:** 2023-11-16

**Authors:** Privilege Tendai Munjoma, Panashe Chandiwana, Jacqueline Wyss, Arthur John Mazhandu, Sebastian Bruno Ulrich Jordi, Rutendo Gutsire, Leolin Katsidzira, Bahtiyar Yilmaz, Benjamin Misselwitz, Kerina Duri

**Affiliations:** ^1^ Immunology Unit, Department of Laboratory Diagnostic and Investigative Sciences, University of Zimbabwe Faculty of Medicine and Health Sciences (UZ-FMHS), Harare, Zimbabwe; ^2^ Department of Visceral Surgery and Medicine, Inselspital, Bern University Hospital, University of Bern, Bern, Switzerland; ^3^ Department for Biomedical Research, Maurice Müller Laboratories, University of Bern, Bern, Switzerland; ^4^ Department of Internal Medicine, University of Zimbabwe Faculty of Medicine and Health Sciences (UZ-FMHS), Harare, Zimbabwe

**Keywords:** fecal calprotectin, microbial translocation, systemic inflammation, HIV, gut microbiota, lactating women, resource limited setting

## Abstract

**Introduction:**

Combination antiretroviral therapy (cART) effectively controls HIV; however, chronic low-level viremia and gut microbiota dysbiosis remain significant drivers of gut and systemic inflammation. In this study, we explored the relationship between gut microbiota composition, intestinal inflammation, microbial translocation, and systemic inflammation in women on cART in Sub-Saharan Africa.

**Methods:**

We conducted a study in HIV-infected and HIV-uninfected lactating women followed up at 6 weeks and 6 months postpartum in Harare, Zimbabwe. We used 16S ribosomal Ribonucleic Acid (rRNA) sequencing and MesoScale Discovery V-Plex assays to examine the gut microbiome and to quantify plasma inflammatory biomarkers, respectively. In addition, we measured fecal calprotectin, plasma lipopolysaccharide-binding protein (LBP), and soluble cluster of differentiation 14 (sCD14) by enzyme-linked immunosorbent assay to assess gut inflammation, microbial translocation, and monocyte/macrophage activation.

**Results:**

A group of 77 lactating women were studied, of which 35% were HIV-infected. Fecal calprotectin levels were similar by HIV status at both follow-up time points. In the HIV-infected group at 6 weeks postpartum, fecal calprotectin was elevated: median (interquartile range) [158.1 µg/g (75.3–230.2)] in women who had CD4+ T-lymphocyte counts <350 cells/µL compared with those with ≥350 cells/µL [21.1 µg/g (0–58.4)], p = 0.032. Plasma sCD14 levels were significantly higher in the HIV-infected group at both 6 weeks and 6 months postpartum, p < 0.001. Plasma LBP levels were similar, but higher levels were observed in HIV-infected women with elevated fecal calprotectin. We found significant correlations between fecal calprotectin, LBP, and sCD14 with proinflammatory cytokines. Gut microbial alpha diversity was not affected by HIV status and was not affected by use of antibiotic prophylaxis. HIV significantly affected microbial beta diversity, and significant differences in microbial composition were noted. The genera *Slackia* and *Collinsella* were relatively more abundant in the HIV-infected group, whereas a lower relative abundance of *Clostriduim sensu_stricto_1* was observed. Our study also found correlations between gut microbial taxa abundance and systemic inflammatory biomarkers.

**Discussion and conclusion:**

HIV-infected lactating women had increased immune activation and increased microbial translocation associated with increased gut inflammation. We identified correlations between the gut inflammation and microbial composition, microbial translocation, and systemic inflammation. The interplay of these parameters might affect the health of this vulnerable population.

## Introduction

People living with HIV in Sub-Saharan Africa (SSA) constitute about 54% of the world’s HIV-infected population (1). The introduction of combination antiretroviral therapy (cART) has reduced the burden of HIV in SSA with a significant reduction in morbidity and mortality. However, chronic persistent low-level viremia due to residual HIV remains a significant contributor to microbial translocation, chronic monocyte activation, and inflammation in this population (2–4). Understanding the relationship between these biological systems contributes to the global effort of interventions toward the mitigation of HIV-associated morbidities.

The acute phase of HIV infection involves depletion of the CD4+ T-cell population, causing major damage to gut-associated lymphoid tissue, which is not fully restored by cART (5, 6). Upon gut inflammation, neutrophils serve as a reliable defense mechanism. Gut inflammation causes increased gut permeability and release of calprotectin from neutrophils. The fecal calprotectin levels are thus a useful marker of gut inflammation and an indirect marker of intestinal permeability (7). Faecal calprotectin levels have been reported to be higher in the HIV-infected population even if on cART compared with that in HIV-uninfected peers (8, 9). Despite these studies, the influence of gut inflammation on microbial translocation and systemic inflammation is still insufficiently understood.

Inflammation of the gut epithelial lining causes translocation of microbial antigens into circulation driving HIV disease progression through monocyte activation and inflammation (10). The monocyte/macrophage bound cluster of differentiation 14 (CD14) is a co-receptor for lipopolysaccharide (LPS) and causes the secretion of soluble CD14 (sCD14) (11) on exposure to bacterial toxins. Thus, both LPS-binding protein (LBP) and sCD14 are considered biomarkers of endotoxemia and intestinal permeability, which also alters the gut microbiota (12). Increased systemic inflammation with accompanied gut permeability has been observed in cART-treated women (13). However, long-term exposure to cART has been shown to decrease biomarkers of gut permeability, microbial translocation, and vascular injury in adults with chronic HIV infection (6, 14, 15).

A healthy gut microbiota is usually dominated by commensal microorganisms that continually face perturbations such as HIV-induced gut damage and antibiotics. Dysbiosis as a result of HIV infection is generally characterized by a decrease in alpha diversity (16, 17) with a low abundance of *Bacteroides* and an increased abundance of *Prevotella* (18). However, further evidence is needed to further understand the impact of HIV-associated gut microbiota dysbiosis on the production of proinflammatory cytokines and the consequent systemic inflammation (19). Microbial antigens translocated into circulation causes immune activation with higher levels being associated with increased T-cell activation in cART-treated individuals (15). In early chronic HIV infection, circulating LPS has been shown to be a predictor of HIV disease progression independent of HIV viremia and CD4+ T-lymphocyte count (20). Evidence of the role of HIV-induced gut microbiota dysbiosis in microbial translocation and inflammation has been conflicting due to possible confounders in HIV management such as antibiotic prophylaxis and cART.

To gain an understanding of the intricate relationships between the processes, we hypothesized that microbial dysbiosis and inflammation of the gut due to HIV infection cause microbial translocation and, ultimately, systemic inflammation. Our study aims to provide insights into the gut microbiota diversity and abundance in HIV-infected and HIV-uninfected lactating women at 6 weeks and 6 months postpartum. Biomarkers of systemic inflammation and their association with gut microbiota abundance, gut inflammation, and microbial translocation were investigated.

## Materials and methods

### Study design

This investigation was performed as a prospective longitudinal study nested in the University of Zimbabwe Birth Cohort Study (UZBCS). The UZBCS has been previously described in detail (21). In brief, lactating women were longitudinally followed up at 6 weeks and 6 months postpartum as part of a longitudinal follow-up to 2 years after birth.

### Study participants

The study followed up women enrolled in the UZBCS who were HIV-infected and HIV-uninfected and receiving postnatal care services. They were monitored at 6 weeks and 6 months after giving birth at four primary healthcare clinics located in areas with low socio-economic status in Harare, Zimbabwe (Budiriro, Glenview, Kuwadzana, and Rujeko clinics).

### Inclusion and exclusion criteria

We recruited pregnant women beyond the 20th week of pregnancy seeking antenatal care services. All participants gave written informed consent to participate and had been tested for HIV. Women who failed to adhere to the study procedures due to any health disorders such as mental issues were not included in this sub-study. For this particular study, only women who enrolled in the UZBCS in 2019 and had stool samples available were included. These women were then followed up at 6 weeks and 6 months after giving birth.

### Data collection, sample collection, and storage

Data were collected using paper-based approved questionnaires and entered into a Research Electronic Data Capture (REDCap) database—a secure, web-based software platform designed to support data capture for research studies (22). A physical examination including anthropometric assessments was carried out by trained and qualified nurses. A total of 4 mL of whole venous blood samples, collected using ethylenediamine-tetraacetic acid as an anticoagulant, were obtained from each participant. The blood collection procedure was carried out by trained and qualified nurses, ensuring adherence to proper protocols. The collected samples were promptly processed within a maximum time frame of 6 h from the time of collection. Plasma was isolated and stored at −80°C until enzyme-linked immunosorbent assays (ELISAs) and MesoScale Discovery (MSD) V-plex assays were performed. About 50 g of feces was collected in sterile containers, aliquoted, and stored at −80°C until fecal calprotectin and DNA extraction assays were done.

### HIV RNA load and CD4+ T-lymphocyte counts

Results of HIV RNA load and CD4+ T-lymphocyte counts measured during the third trimester of pregnancy were obtained from the UZBCS REDCap database. The assaying methods for these HIV disease progression markers were previously described (21). All HIV-infected women in this study were taking cART at both follow-up time points, and used a formulation of efavirenz, lamivudine, and tenofovir disoproxil fumarate (Tenolam-E), following the World Health Organization guidelines (23).

### Systemic inflammation and immune biomarkers

The MSD multi-spot V-plex assays (Rockville, Maryland, USA) were used to quantify proinflammatory and vascular injury immune markers in 60 µL of plasma. The assays were carried out following the manufacturer’s instructions and as previously described (24). Originally, 48 biomarkers were quantified in plasma, and, for this study, the proinflammatory and vascular injury V-plex panels were of interest. The proinflammatory V-plex panel included interferon-gamma (IFN-γ), interleukin-1-beta (IL-1β), interkeukin-2 (IL-2), interleukin-4 (IL-4), interleukin-6 (IL-6), interleukin-8 (IL-8), interleukin-10 (IL-10), interleukin-12p70 (IL-12p70), interleukin-13 (IL-13), and tumor necrosis factor (TNF), and the vascular injury V-plex panel included serum amyloid A (SAA), C-reactive protein (CRP), vascular cell adhesion molecule 1 (VCAM-1), and intercellular Adhesion Molecule 1 (ICAM-1). The biomarker SAA was excluded from our analyses due to calibration failure in one of the assays.

### Biomarker of gut inflammation

Fecal calprotectin was quantified from stool using a Buhlmann fecal calprotectin sandwich ELISA assay (EK-CAL2-WEX, Schönenbuch, Switzerland) based on the manufacturer’s instructions. The Buhlmann Calex Cap (B-CALEX-C200, Schönenbuch, Switzerland) was utilized to prepare stool extracts following the manufacturer’s instructions. The extracts were then diluted at a ratio of 1:5 with an appropriate incubation buffer before proceeding with the assay. All assays were conducted in duplicate, and ELISA plates were read at 450 nm using Gen 5 software (BioTek, Winooski, VT, USA). Fecal calprotectin concentrations were determined using a standard curve and categorized based on the manufacturer’s clinical cutoffs: normal (<80 µg/g), borderline/grey zone (80–160 µg/g), and elevated (>160 µg/g).

### Biomarkers of microbial translocation and monocyte activation

Plasma LBP and sCD14 levels were quantified using ELISA assays (Hycult Biotech, Wayne, USA) according to the manufacturer’s instructions. Absorbance was read at 450 nm, and sample concentrations were determined from a standard curve. All assays were performed in duplicate, and ELISA plates were read using Gen 5 software (BioTek, Winooski, VT, USA).

### Stool DNA Extraction and 16S rRNA sequencing

Fecal samples were collected into sterile 50-mL sample cups, aliquoted into 2-mL tubes, and stored at −80°C prior to assays. Fecal DNA extraction was carried out from about 250 mg of stool sample using the QIAamp PowerFecal Pro DNA kit (Qiagen, Dusseldorf, Germany) as previously described (25). Total bacterial DNA was eluted with 70 μL of elution buffer and then stored at −20°C prior to PCR amplification. The eluted DNA was amplified using PCR, targeting the V5 and V6 regions of the 16S rRNA gene. Previously described bacteria-specific primers (forward: 5′-CCATCTCATCCCTGCGTGTCTCCGACTCAGC-barcode-ATTAGATACCCYGGTAGTCC-3′ and reverse: 5′-CCTCTCTATGGGCAGTCGGTGATA CGAGCTGACGACARCCATG-3′) were utilized (26). PCR conditions consisted of an initial denaturation at 94°C for 5 min, followed by 35 cycles of denaturation at 94°C for 1 min, annealing at 46°C for 20 s, elongation at 72°C for 30 s, and a final elongation at 72°C for 7 min.

The PCR amplicons were run on 1% agarose gel electrophoresis at 100 volts for 1 h, with an expected product length of approximately 350 base pairs. The amplicons were purified using the QIAQuick Gel Extraction Kit (Qiagen, Dusseldorf, Germany). The concentration of amplicons was determined using a Qubit dsDNA HS Assay Kit on the Qubit 3.0 Fluorometer (ThermoFisher Scientific) and then set to 26 pM for sequencing library preparation. Sequencing was carried out on the Ion PGM™ System (ThermoFisher Scientific) using an Ion PGM™ Sequencing kit and chip, following a previously described method (27).

## Data analysis

### Sociodemographic and participant characteristics

Data analysis was conducted using R software version 4.2.2 (http://www.r-project.org/). Continous variables were tested for normality using the Shapiro–Wilk test, and data were summarized using median and interquartile range (IQR) or using mean ± standard deviation (SD) where appropriate. Continous data between groups were compared using the Mann–Whitney U-test, Kruskal–Wallis test, or Student T-test depending on the distribution of the data. Categorical data were reported as proportions and associations determined by Fisher’s exact test or Chi-squared test where appropriate.

### Microbial translocation, monocyte activation, gut inflammation, and systemic inflammatory bıomarkers

Continous data were tested for normalcy using the Shapiro–Wilk test and data reported as median (IQR) or mean ± SD where appropriate, depending on data distribution. Concentrations of proinflammatory and vascular injury immune markers below the assay detection limit were assigned the concentration of the lowest calibrator as previously described (24). A q-value was calculated to correct for multiple testing using the Bonferroni test, and q < 0.05 was considered significant. Correction was done for the 48 biomarkers originally tested during the assays. Spearman rho (ρ) correlation coefficient was used to determine associations between biomarkers of HIV disease progression, gut inflammation, microbial translocation, and systemic inflammation.

### Computational analysis of 16S rRNA microbial data

The Fastq sequencing files generated from Ion Torrent PGM™ System were processed using the Quantitative Insights into Microbial Ecology 2 (QIIME2) version 2021.11.0 pipeline (https://qiime2.org/), as previously described (26, 28, 29). Amplicon sequence variants were assigned with a 97% sequence identity threshold, using the default options in QIIME2 as well as the *q2-feature-classifier* plugin and a Naïve Bayes classifier. Taxonomic weights were assembled using the SILVA database (https://www.arb-silva.de/).

The feature table and mapping file were used to generate a phyloseq object in R (version 4.2.2) package *phyloseq* (30). Only samples with more than 2,000 high-quality reads were further analyzed. Diversity within communities was determined using alpha diversity indices (Simpson and Shannon index), and inter-community diversity was determined using beta diversity [Bray–Curtis dissimilarity using principal coordinate analysis (PCoA)] (31). Mann–Whitney U-tests and Adonis (PERMANOVA) tests for alpha diversity and beta diversity were performed to test for significance of any group differences, respectively. Taxonomy profiling was performed using microbiome Multivariable Association with Linear Models (MaAsLin2) package (https://huttenhower.sph.harvard.edu) to determine associations of the gut microbiota with categorical and continuous variables (32). A q-value was calculated to correct for multiple testing using the Benjamini–Hochberg (BH) false discovery rate (FDR) correction as a default step in MaAsLin2. A q-value <0.05 was considered significant, and microbiota plots were generated using the package *phyloseq* and GraphPad prism version 9.0.0 (GraphPad, San Diego, CA).

## Results

Of the 97 women enrolled since 2019, 77 lactating women were successfully followed up at both 6 weeks and 6 months postpartum. Maternal socio-demographics, concurrent medications, and clinical characteristics at 6 weeks postpartum are shown in **Table 1**. The HIV-infected women [median age, 32 years (IQR, 29–35)] were older than the HIV-uninfected counterparts [median age, 26 years (IQR, 21–30)].

**Table 1:** Socio-demographic and clinical data of the study participants at 6 weeks postpartum (n **=** 77), stratified by HIV status.

	HIV-infected (n = 27)	HIV-uninfected (n = 50)	p-value
Social demographics			
**Age (years)** [Median (IQR)]	32 (29–35)	26 (21–30)	**3.95e-05**
**Breastfeeding type** Exclusive Mixed	25 (92.6%) 2 (7.4%)	42 (84%) 8 (16%)	0.479
**Postpartum alcohol use** Yes No	1 (3.7%) 26 (96.3%)	1 (2%) 49 (98%)	1.000
**Household meals per day** [Median (IQR)]	3 (2-3)	3 (2-3)	0.569
**Average stool frequency** Once daily Greater or equal to twice daily Once every 2 days	21 (77.8%) 4 (14.8%) 2 (7.4%)	37 (75.5%) 9 (18.4%) 3 (6.1%) Missing=1	0.883
Concurrent medications			
**Antibiotics use** Yes No	15 (55.6%) 12 (44.4%)	2 (4%) 48 (96%)	**4.48e-07**
**Anti-acid use** Yes No	0 (0%) 27 (100%)	3 (6%) 47 (94%)	0.547
**cART use** Yes No	27 (100%) 0 (0%)	NA	_
Clinical data			
**MUAC (cm)** [Median (IQR)]	27.0 (25.7–28.0)	25.4 (24.0–27.3)	**0.031**
**BMI (kg/m^2^)** [Median (IQR)]	24.2 (22.1–25.6)	22.1 (19.6–23.9)	**0.020**
**Mode of delivery** Spontaneous Caesarean section	24 (88.9) 3 (11.1%)	50 (100%) 0 (0%)	**0.039**
**cART duration (months)** [Median (IQR); min-max]	40.8 (13.5–86.1); 1.8–1455.4	NA	_
**cART duration group** Early ART (<2 years) Long-term ART (≥2 years)	8 (29.6%) 19 (70.4%)	NA	_
**Third trimester CD4 count (cells/µL)** [Median (IQR); min-max]	355 (253–449); 176–635	NA	_
**Third trimester HIV RNA suppression** Suppressed (≤1,000 copies/mL) Unsuppressed (>1,000 copies/mL)	23 (85.2%) 4 (14.8%)	NA	_
**Third trimester HIV Viremia** **Low-level** (<200 copies/mL) **Viremic** (≥200 copies/mL)	23 (85.2%) 4 (14.8%)	NA	_

BMI, body mass index; MUAC, mid-upper–arm circumference; IQR, interquartile range; HIV, human immunodeficiency virus; CD4, cluster of differentiation 4; cART, combination anti-retroviral therapy; RNA, ribonucleic acid. **Statistical analysis:** Group comparisons were done using Mann–Whitney U-test or Fisher’s exact test where appropriate. P-values in bold font are statistically significant at p < 0.05.

At 6 weeks postpartum, all HIV-infected women were on cART, and 15 (55.6%) were taking cotrimoxazole prophylaxis. In all these women, cART was started pre-conception or during pregnancy. HIV-infected women were more likely to be on antibiotics during the study period, p < 0.0001. In addition, HIV-infected women had a significantly higher mid-upper–arm circumference (MUAC) (27.0 cm; IQR, 25.7–28.0) compared with their HIV-uninfected peers (25.4 cm; IQR, 24.0–27.3), p = 0.031. Interestingly, HIV-infected women had a higher body mass index (BMI) compared with their uninfected counterparts (p = 0.020), as indicated in **Table 1**.

Socio-demographics and clinical characteristics at 6 months postpartum are shown in **Table 2**. HIV-infected women were more likely to be in employed and to have a higher median monthly family income (**Table 2**). There were no associations between HIV infection status and toilet facilities, drinking water sources, and water treatment. Similar to the 6-week postpartum findings, HIV-infected women were more likely to be on antibiotics (see **Table 1**). In the HIV-infected group 55.6% and 57.1% reported taking cotrimoxazole antibiotics at 6 weeks and 6 months postpartum, respectively.

**Table 2:** Socio-demographic, water, hygiene and sanitation and concurrent medications at 6 months postpartum (n = 77), stratified by HIV status.

	HIV-infected (n = 28)	HIV- uninfected (n = 49)	p-value
Social demographics			
**Employment status** Employed Unemployed	14 (50%) 14 (50%)	11 (22.4%) 38 (77.6%)	**0.044**
**Household size** [Median (IQR)]	5 (4–5)	4 (3–5)	0.074
**Family monthly income (USD)** [Median (IQR)]	1,150 (800–1,450)	650 (480–1,285)	**0.017**
Water, hygiene and sanitation			
**Toilet facility in household** Flush (outside/inside) Blair	27 (96.4%) 1 (3.6%)	47 (95.9%) 2 (4.1%)	0.155
**Households sharing toilet** [Median (IQR)]	1 (1–4)	3 (2–4)	0.106
**Main drinking water source** Borehole Piped water into dwelling Protected well	18 (64.3%) 1 (3.6%) 9 (32.1%)	22 (44.9%) 5 (10.2%) 22 (44.9%)	0.293
**Drinking water treatment** No Yes	21 (75%) 7 (25%)	42 (85.7%) 7 (14.3%)	0.357
**Current sewer burst/overspill** No Yes	25 (89.3%) 3 (10.7%)	41 (85.4%) 7 (14.6%) Missing = 1	0.736
**Current diarrhea (participant/household member)** No Yes	26 (92.9%) 2 (7.1%)	42 (89.4%) 5 (10.6%) Missing = 2	0.705
**Average stool frequency** Once daily Greater or equal to twice daily Once every 2 days	23 (82.1%) 4 (14.3%) 1 (3.6%)	34 (69.4%) 14 (28.6%) 1 (2%)	0.357
Concurrent medications			
**Antibiotics use** Yes No	16 (57.1%) 12 (42.9%)	0 (0%) 49 (100%)	**2.23e-09**
**Anti-acid use** Yes No	0 (0%) 27 (100%) Missing = 1	0 (0%) 49 (100%)	_

IQR, interquartile range; HIV, human immunodeficiency virus. **Statistical analysis:** Group comparisons were done using the Mann–Whitney U-test or Fisher’s exact test where appropriate. P-values in bold font are statistically significant at p < 0.05.

### Biomarkers of gut inflammation and gut microbial translocation

Biomarkers of intestinal inflammation (fecal calprotectin), microbial translocation, and immune activation (plasma LBP and sCD14) were quantified to assess the association between HIV infection with gut inflammation and microbial gut translocation. Overall, fecal calprotectin levels did not significantly differ by HIV infection status and antibiotic use at 6 weeks and 6 months postpartum. However, at 6 weeks, higher fecal calprotectin levels (158.1 µg/g; IQR, 75.3–230.2) were observed in HIV-infected women with third-trimester CD4+ T-lymphocyte counts <350 cells/µL compared with those with ≥350 cells/µL (21.1 µg/g; IQR, 0–58.4), p = 0.032. In a sub-analysis of the HIV infected at 6 weeks postpartum, we investigated the effects of cART and HIV viremia. We stratified cART duration into early (<2 years) and long-term (≥2 years) (33) and HIV RNA load into low level viremia (<200 copies/mL) and viremic (≥200 copies/mL) groups (4). Fecal calprotectin levels were non-significantly higher in unsuppressed (HIV RNA > 1,000 copies/mL) versus suppressed (≤1,000 copies/mL) in viremic versus low level viremia groups (p = 0.610 and p = 0.614, respectively). Levels were non-significantly higher (116.7 µg/g; IQR, 43.3–230) in the early ART group compared with that in the long-term ART group (61.3 µg/g; IQR, 16.3–165.3).

**Figure 1:**
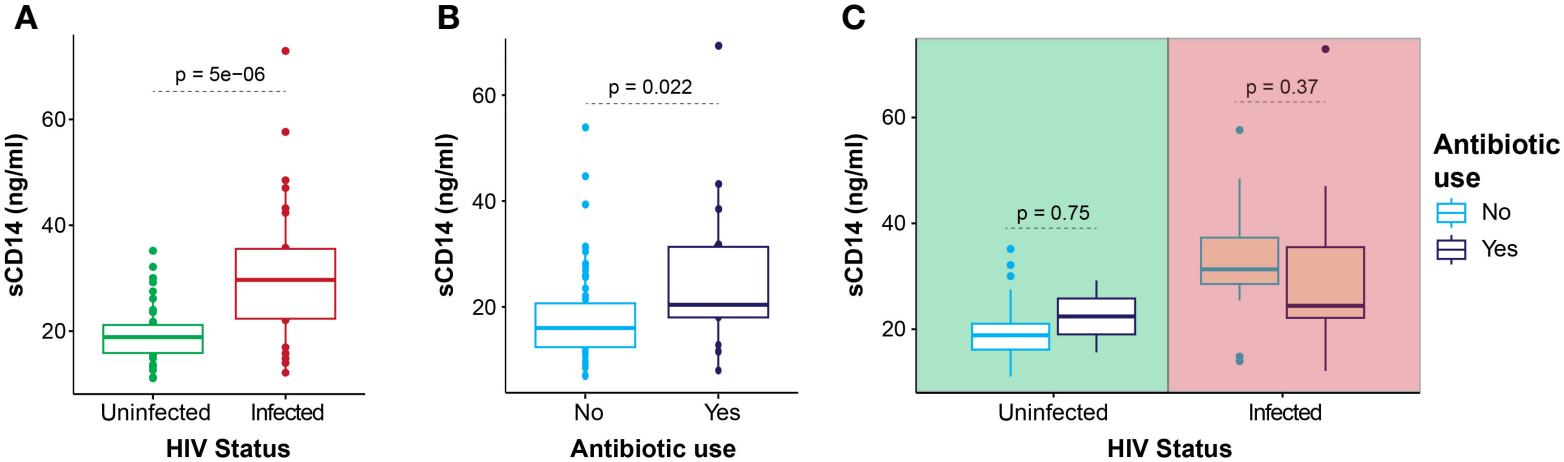
Plasma sCD14 levels. Plasma sCD14 levels in all participants at 6 weeks postpartum, according to HIV infection status **(A)**, antibiotic use **(B)**, and stratified for HIV status **(C)**. Statistics: Mann–Whitney U-test.

HIV-infected participants had significantly higher plasma sCD14 levels (29.7 ng/mL; IQR, 22.4–35.5) at 6 weeks postpartum when compared with their uninfected counterparts (18.9 ng/mL; IQR, 15.9–21.2), p < 0.0001 (**Figure 1A**). Similar results were found at 6 months postpartum where increased sCD14 levels were found in HIV-infected (33.2 ng/mL; IQR, 25.0–36.2) compared with that in HIV-uninfected women (22.7 ng/mL; IQR, 19.0–27.0), p = 0.0006.

In the HIV-infected group at 6 weeks postpartum, sCD14 levels were similar when compared by cART duration and HIV viremia groups (p = 0.449 and p = 0.921 respectively). In all women, at 6 weeks postpartum, those taking antibiotics had significantly higher plasma sCD14 levels (24.4 ng/mL; IQR, 22.1–35.3) (**Figure 1B**) compared with those not taking antibiotics (20.1 ng/mL; IQR, 16.5–24.7). Plasma sCD14 levels did not differ by antibiotic use in the subgroups of HIV-infected and HIV-uninfected women (**Figure 1C**). At 6 months postpartum, only HIV-infected women were on regular antibiotics, and no significant difference in median sCD14 levels was noted on comparison by antibiotic usage. Furthermore, plasma sCD14 levels at 6 weeks and 6 months postpartum did not differ between women with elevated and normal fecal calprotectin levels.

**Figure 2:**
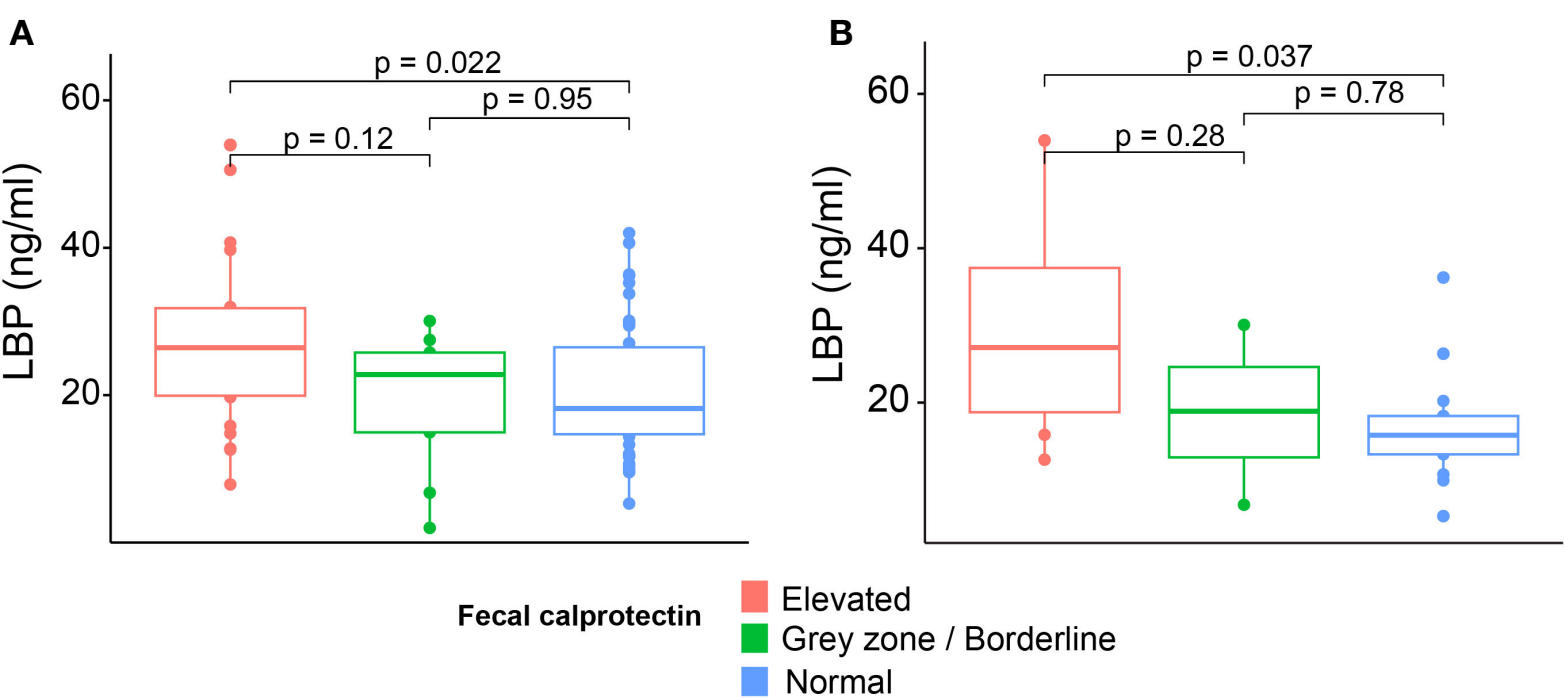
Plasma LBP levels by fecal calprotectin group. Plasma LBP levels in all participants **(A)** and HIV-infected women **(B)** at 6 weeks postpartum stratified for fecal calprotectin group. Statistics: Mann–Whitney U-test.

Plasma LBP levels were similar between the HIV subgroups at 6 weeks or 6 months postpartum. In the HIV-infected group at 6 weeks postpartum, LBP levels were similar when compared by cART duration and HIV viremia groups (p = 0.632 and p = 0.453, respectively). At the same time point, most HIV-infected women (88.2%) were on cotrimoxazole prophylaxis, and there was no significant difference in plasma LBP levels by antibiotic use. At 6 weeks postpartum, significantly lower LBP levels were noted in women with normal fecal calprotectin levels compared with those with elevated levels [18.2 ng/mL (IQR, 14.7–26.5) versus 26.4 ng/ml (IQR, 19.9–31.8), p = 0.022] (**Figure 2A**). A similar trend was observed in the HIV-infected subgroup at the same time point (**Figure 2B**).

### Correlation between biomarkers of gut inflammation, microbial translocation, and systemic inflammation

**Figure 3:**
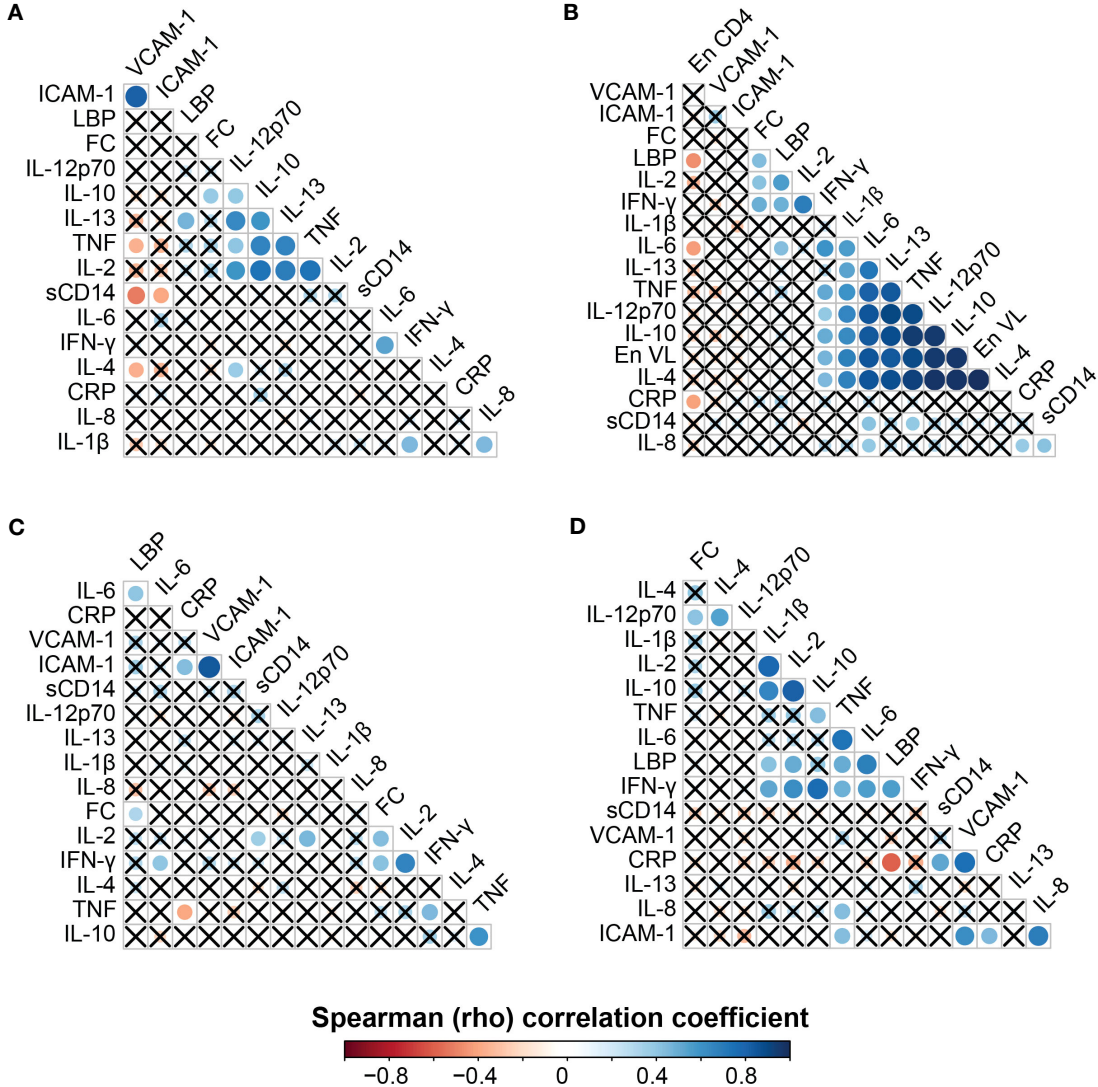
Correlation matrix analysis of plasma biomarkers in HIV-infected and HIV-uninfected women at 6 weeks and 6 months postpartum. HIV-uninfected participants at 6 weeks postpartum **(A)**, HIV-infected participants at 6 weeks postpartum **(B)**, HIV-uninfected participants at 6 months postpartum **(C)**, and HIV-infected participants at 6 months postpartum **(D)**. Correlations marked by X were either not present or non-significant at p < 0.05.

To determine whether biomarkers of gut inflammation (fecal calprotectin) and microbial translocation (sCD14 and LBP) are associated with the systemic immune environment, a correlation matrix of these biomarkers and 13 proinflammatory cytokines and chemokines was computed (**Figure 3**). These correlations were calculated after stratification by HIV status to minimize confounding by known effects of HIV on the cytokine and chemokine environment.

In the HIV-uninfected women, at 6 weeks postpartum, fecal calprotectin and LBP correlated positively with IL-10 (ρ = 0.38, p = 0.0376) and IL-13 (ρ = 0.47, p = 0.0082), respectively (**Figure 3A**). In the same group at 6 months postpartum, LBP correlated positively with IL-6 (ρ = 0.40, p = 0.024) and fecal calprotectin (ρ = 0.31, p = 0.032), whereas fecal calprotectin correlated positively with IL-2 (ρ = 0.43, p = 0.018) and IFN-γ (ρ = 0.42, p = 0.022). The biomarker sCD14 correlated positively with IL-2 (ρ = 0.36, p = 0.046) in this subgroup at 6 months postpartum (**Figure 3C**).

In the HIV-infected group, pregnancy third-trimester CD4+ T-lymphocyte count (En CD4) and pregnancy third-trimester HIV RNA load (En VL) were included in the correlation analysis at 6 weeks postpartum (**Figure 3B**). We assumed that the time since the third trimester of pregnancy may have caused insignificant changes in CD4+ T-lymphocyte counts and HIV RNA load. Interestingly, third-trimester CD4+ T-lymphocyte counts negatively correlated with LBP (ρ = −0.47, p = 0.019), IL-6 (ρ = −0.43, p = 0.033) and CRP (ρ = −0.42, p = 0.035). Third-trimester HIV RNA levels (En VL) positively correlated with eight proinflammatory cytokines and chemokines, p < 0.05 (**Figure 3B**). In the HIV-infected group at 6 weeks postpartum, fecal calprotectin correlated positively with LBP (ρ = 0.44, p = 0.027), IL-2 (ρ = 0.42, p = 0.037), and IFN-γ (ρ = 0.49, p = 0.012), whereas LBP correlated positively with IL-2 (ρ = 0.56, p = 0.002), IFN-γ (ρ = 0.48, p = 0.010), and IL-6 (ρ = 0.43, p = 0.026).

At 6 weeks postpartum, sCD14 levels positively correlated with IL-8 (ρ = 0.42, p = 0.029), TNF (ρ = 0.39, p = 0.044), and IL-6 (ρ = 0.42, p = 0.029) in the HIV-infected group (**Figure 3B**). At 6 months postpartum, fecal calprotectin positively correlated with IL-12p70 (ρ = 0.41, p= 0.044) in the HIV-infected group (**Figure 3D**), whereas LBP levels positively correlated with IL-2 (ρ = 0.50, p = 0.010), IL-1β (ρ = 0.42, p = 0.037), IL-6 (ρ = 0.68, p = 0.0002), TNF (ρ = 0.50, p = 0.011), and IFN-γ (ρ = 0.55, p = 0.004). In the same group, plasma sCD14 levels positively correlated with CRP (ρ = 0.52, p = 0.006) (**Figure 3D**).

### Comparison of biomarkers of systemic inflammation and vascular injury according to HIV infection status and follow-up time point

To explore the impact of HIV infection on the cytokine/chemokine environment, we conducted a comparative analysis of median biomarker levels between HIV-infected and HIV-uninfected women at 6 weeks and 6 months postpartum (**Supplementary Figure 1**). We did not observe any statistically significant differences in the biomarker levels between the HIV-infected and HIV-uninfected women even after applying multiple testing correction to account for potential false positives.

### Gut microbiota assessment at 6 weeks and 6 months postpartum

**Figure 4:**
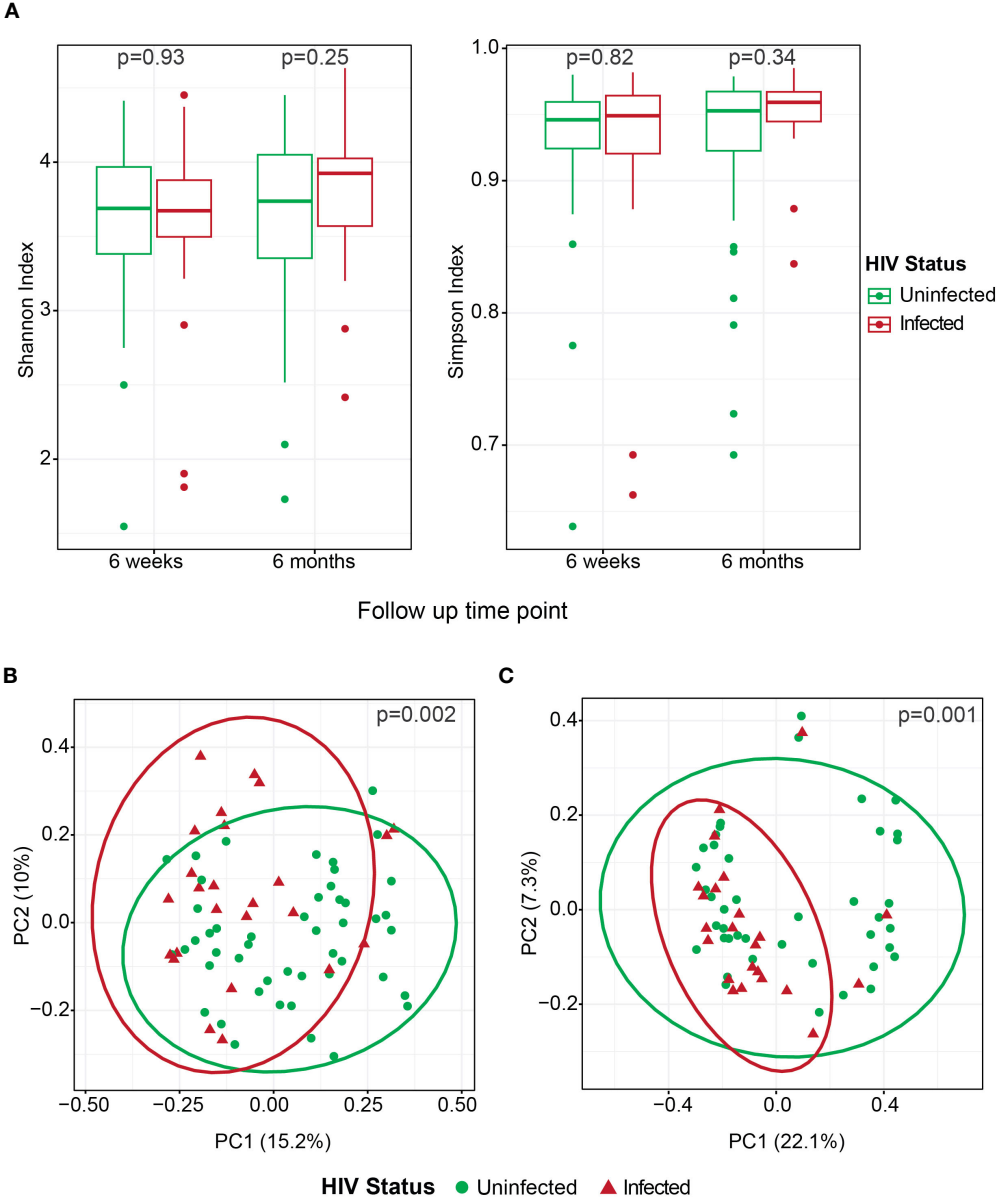
Microbiota characteristics at 6 weeks and 6 months postpartum according to HIV status. Alpha diversity (Shannon and Simpson index) comparison by HIV status at both 6 weeks and 6 months postpartum **(A)**, PCoA for beta diversity (Bray–Curtis) comparison by HIV infection status at 6 weeks postpartum **(B)**, and PCoA for beta diversity (Bray-Curtis) comparison by HIV status at 6 months postpartum **(C)**.

For gut microbiota analysis, samples from 73 of the 77 participants at 6 weeks and 65 of the 77 participants at 6 months postpartum could be used. There were four participants with missing stool samples at 6 weeks and five participants with missing stool samples at 6 months. Seven samples were dropped from analysis due to low sequence read numbers. Overall, alpha diversity measures (Shannon and Simpson index) did not differ significantly between the two follow-up time points or by HIV status (**Figure 4A**). However, there was a significant difference in beta diversity in all participants between the two time points (p = 0.001). Beta diversity also differed significantly by HIV status at 6 weeks (**Figure 4B**) and 6 months (**Figure 4C**) postpartum.

We conducted a comparative analysis of the gut microbiota in the HIV-infected group at 6 weeks postpartum, considering third-trimester HIV RNA suppression (cutoff of ≤1,000 copies/mL) and third-trimester CD4+ T-lymphocyte status (cutoff of ≤350 cells/µL). There were no significant differences in the alpha diversity (assessed by Shannon and Simpson indices) and beta diversity according to HIV RNA suppression and CD4+ T-lymphocyte status. In addition, there were no significant differences in either alpha or beta diversity stratified by antibiotic use in the HIV-infected group at 6 weeks and 6 months postpartum (data not shown). All HIV-infected participants in our study were receiving cART; therefore, the effects of HIV and cART on the gut microbiota could not be separated.

**Figure 5:**
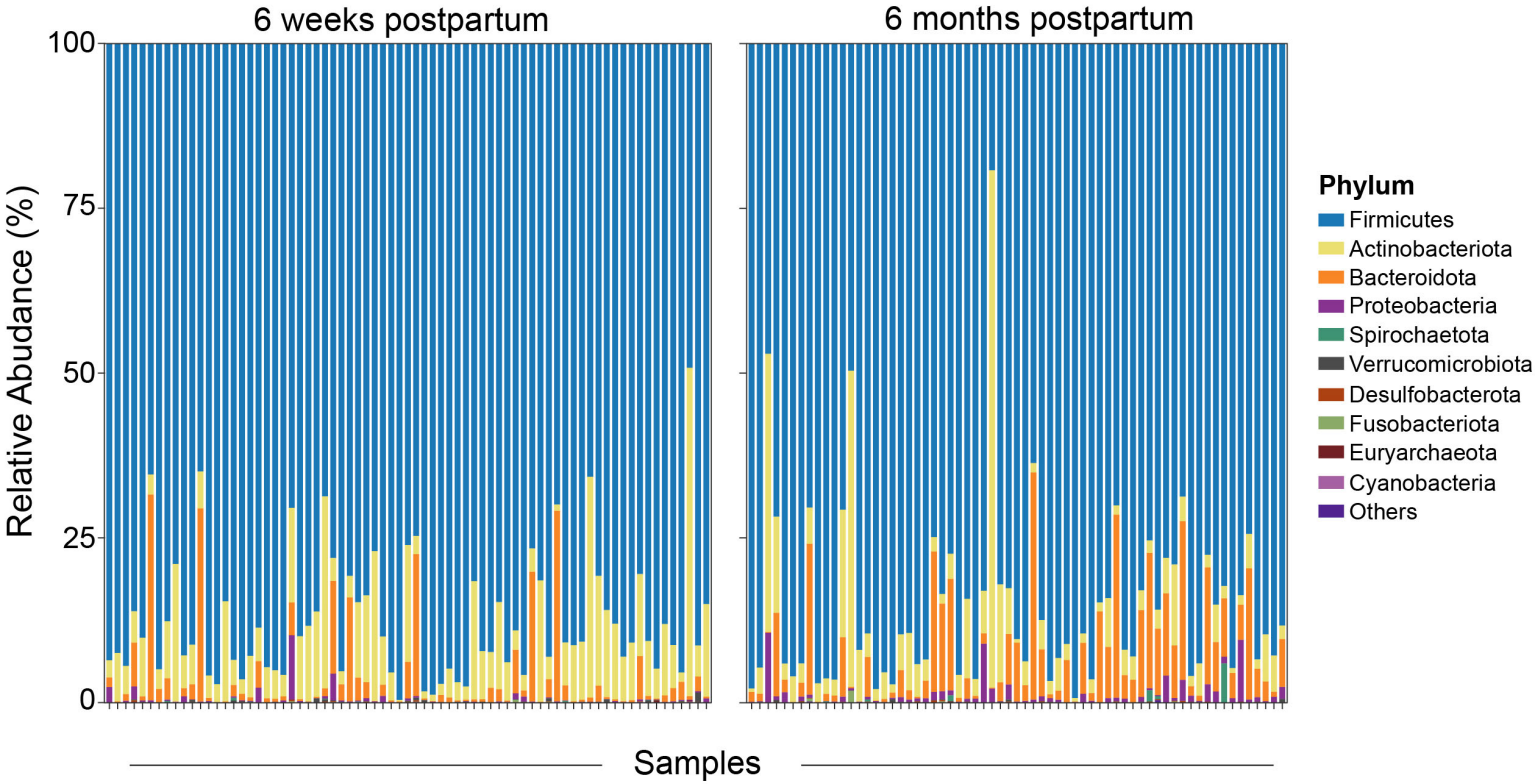
Overall relative abundance for the top 10 phyla of the gut microbiota. The top 10 phyla by order of decreasing relative abundance at both 6 weeks and 6 months postpartum are indicated.

Our study examined the mean relative abundance of the top 10 phyla of the gut microbiota in all women (**Figure 5**). The predominant phyla identified were Firmicutes (86.4%), Actinobacteriota (7.6%), Bacteroidota (4.9%), Proteobacteria (0.8%), Spirochaetota (0.08%), Verrucomicrobiota (0.06%), and Desulfobacterota (0.04%). In addition to that, the mean relative abundance of the top 10 genera was also determined. The predominant genera were *Clostriduim_sensu_stricto_1* (16.6%), *Romboutsia* (16.1%), *Agathobacter* (5.3%), *Faecalibacterium* (4.9%), *Prevotella* (4.4%), *Sarcina* (4.4%), and *Blautia* (3.8%). Notably, in the HIV-infected group, a lower relative abundance of the genera *Akkermansia* and *Collinsella* was noted at 6 months postpartum compared with that at 6 weeks postpartum. However, in the same group, a higher relative abundance of the genera *Lachnospira*, *Prevotella*, *Bacteroides*, and *UCG.005* from the Oscillospiraceae family was noted at 6 months compared with that at 6 weeks postpartum. These findings highlight dynamic shifts in the relative abundance of specific taxa in the postpartum period.

**Figure 6:**
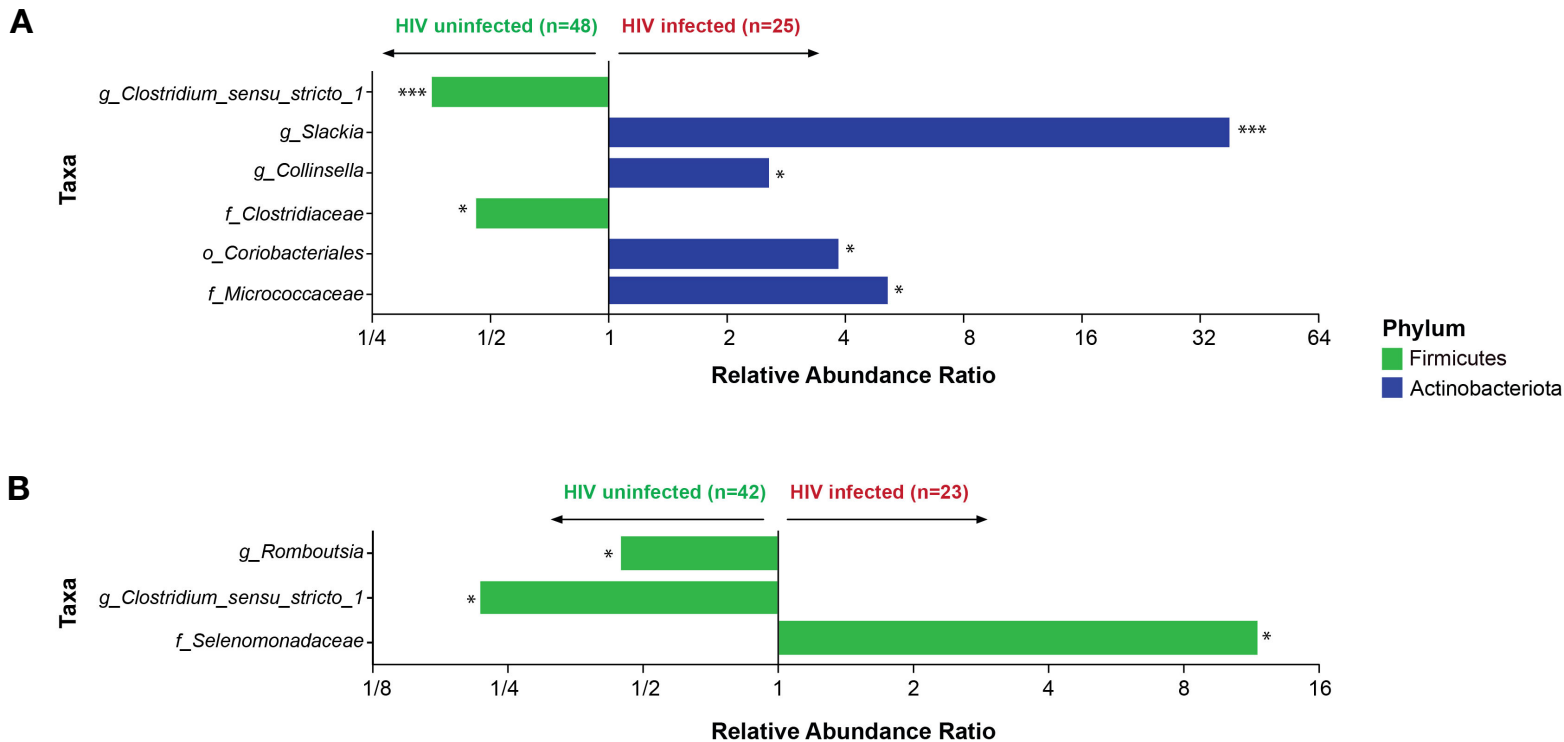
Association of bacterial taxa with HIV infection status. **(A)** Comparisons at 6 weeks postpartum and **(B)** 6 months postpartum are shown. *p < 0.05 and ***p < 0.001.

The gut microbiota was compared to determine taxa significantly associated with HIV infection status at both 6 weeks and 6 months postpartum. At 6 weeks postpartum, HIV infection was significantly associated with lower abundance of genus *Clostriduim_sensu_stricto_1* and family Clostridiaceae from the Firmicutes phylum (**Figure 6A**). At the same time point, HIV infection was significantly associated with a higher abundance of taxa from the Actinobacteriota phylum including the genera *Slackia* and *Collinsella* (**Figure 6A**). At 6 months postpartum, only taxa from the Firmicutes phylum significantly differed by HIV infection status (**Figure 6B**). The genus *Clostriduim_sensu_stricto_1* was significantly abundant in the HIV-uninfected group at both 6 weeks and 6 months postpartum.

We investigated longitudinal changes in the gut microbiota from pregnancy to 6 months postpartum. The pregnancy gut microbiota from our study population has been previously described in detail (34). In brief, species richness was lower (Shannon, p = 0.0092, and Simpson, p = 0.012) in the HIV-infected women compared with that in the uninfected peers. Beta diversity assessed using Bray–Curtis dissimilarity index showed significant differences in diversity between HIV-infected and HIV-uninfected pregnant women. Alpha diversity did not differ by CD4+ T-lymphocyte group and viral load suppression using similar cutoffs used in our study. Infection with HIV was associated with reduced abundance of *Clostridium*, *Bacteroides*, *Bifidobacterium*, and *Faecalibacterium* with an observed increase in *Actinomyces*.

In HIV-uninfected women, there were no differences in microbial evenness between pregnancy and the two follow-up time points (**Supplementary Figure 2A**). However, a significance was noted in microbial richness (p = 0.017) when comparing the three time points. In the HIV-infected group, both evenness and richness indices showed an increase from pregnancy to 6 months postpartum although the difference was non-significant for evenness (**Supplementary Figure 2B**). Beta diversity comparison showed a significant difference (p = 0.001) when compared by time point in HIV-uninfected (**Supplementary Figure 2C**) and HIV-infected groups, p = 0.001 (**Supplementary Figure 2D**). Furthermore, the effects of cART duration and HIV viremia on the gut microbiota in the HIV-infected group were investigated at 6 weeks postpartum. No differences were observed for both Alpha and Beta diversity when compared between early versus long-term cART groups and low-level viremia versus viremic groups.

### Association of inflammatory and vascular injury biomarkers with the gut microbiota abundance

We determined the association of the gut microbiota with inflammatory and vascular injury biomarkers after stratification by HIV infection status using the MaAsLin2 package in R. Only associations remaining significant (q < 0.05) after BH FDR correction were reported (**Table 3**). To test for microbial taxa associations with proinflammatory and vascular injury biomarkers, microbial features that appeared in ≥25% of the total number of samples assayed. In the HIV-infected group at 6 months postpartum, the genera *Catenibacterium* and *Haemophilus* positively associated with levels of IL-2 and IL-6 respectively (q = 0.021 and q = 0.017, respectively).

**Table 3:** Gut microbial taxa and systemic inflammation.

Feature	Metadata	Coefficient	N	N not 0%	q-value
HIV-infected (6 weeks postpartum)					
p:Proteobacteria.c:Gammaproteobacteria.o:Burkholderiales.f:Oxalobacteraceae	CRP	−0.6080931	25	4	0.023359
p:Firmicutes.c:Clostridia.o:Lachnospirales.f:Lachnospiraceae.g:Eubacterium_eligens_group	ICAM-1	0.43383237	25	3	0.000235
HIV-uninfected (6 weeks postpartum)					
p:Firmicutes.c:Bacilli.o:Lactobacillales.f:Leuconostocaceae.g:Weissella	IL-4	0.9326621	48	9	6.04e-08
HIV-infected (6 months postpartum)					
p:Firmicutes.c:Bacilli.o:Erysipelotrichales.f:Erysipelatoclostridiaceae.g:Catenibacterium	IL-2	1.32753279	23	9	0.021138
p:Proteobacteria.c:Gammaproteobacteria.o:Pasteurellales.f:Pasteurellaceae.g:Haemophilus	IL-6	1.58409509	23	6	0.017096
p:Firmicutes.c:Clostridia.o:Lachnospirales.f:Lachnospiraceae.g:Eubacterium_eligens_group	IL-2	0.55998378	23	4	0.001936
p:Firmicutes.c:Clostridia.o:Lachnospirales.f:Lachnospiraceae.g:Eubacterium_eligens_group	IL-10	0.60010197	23	4	0.000557
p:Firmicutes.c:Clostridia.o:Lachnospirales.f:Lachnospiraceae.g:Anaerostignum	IL-4	0.45052327	23	3	0.017532
p:Firmicutes.c:Clostridia.o:Lachnospirales.f:Lachnospiraceae.g:Butyrivibrio	IL-6	0.76872521	23	3	4.03e-05
p:Firmicutes.c:Clostridia.o:Lachnospirales.f:Lachnospiraceae.g:Butyrivibrio	TNF	0.70911458	23	3	0.001431
HIV-uninfected (6 months postpartum)					
p:Actinobacteriota	LBP	0.83076488	42	42	0.018545
p:Firmicutes	IL-2	−0.1308627	42	42	0.041327
p:Actinobacteriota.c:Coriobacteriia.o:Coriobacteriales.f:Coriobacteriaceae	IL-2	0.91371356	42	41	0.029222
p:Firmicutes.c:Clostridia.o:Clostridia_UCG.014.f:Clostridia_UCG.014.g:Clostridia_UCG.014	IFN-γ	−1.5113457	42	36	0.033319
p:Actinobacteriota.c:Actinobacteria.o:Actinomycetales.f:Actinomycetaceae.g:Actinomyces	FC	0.97433139	42	32	0.03262
p:Firmicutes.c:Bacilli.o:Erysipelotrichales.f:Erysipelatoclostridiaceae	ICAM-1	2.02529785	42	27	0.033465
p:Firmicutes.c:Bacilli.o:Erysipelotrichales.f:Erysipelotrichaceae.g:Solobacterium	ICAM-1	1.74957426	42	12	0.033465
p:Actinobacteriota.c:Coriobacteriia.o:Coriobacteriales.f:Eggerthellaceae.g:Eggerthella	FC	1.21725587	42	9	0.03262
p:Actinobacteriota.c:Coriobacteriia.o:Coriobacteriales.f:Eggerthellaceae.g:Eggerthella	IL-2	1.3634006	42	9	0.0208
p:Firmicutes.c:Bacilli.o:Lactobacillales.f:Lactobacillaceae.g:Lactobacillus	IL-8	1.04516168	42	9	0.041506
p:Fusobacteriota.c:Fusobacteriia.o:Fusobacteriales.f:Fusobacteriaceae	FC	1.30098884	42	6	0.001424

Association between gut microbial taxa and microbial translocation, and systemic and gut inflammation biomarkers. N = total number of samples used in model; N not 0% = number of samples in which microbial feature is not 0%; FC, fecal calprotectin.

In the HIV-uninfected group at 6 months, the Actinobacteriota phylum positively associated with LBP levels (q = 0.018), whereas the Firmicutes phylum negatively associated with IL-2 levels (q = 0.041). The family Coriobacteriaceae positively associated with IL-2 levels (q = 0.029), and the genus *Clostridia_UCG.014* negatively associated with IFN-γ levels (**Table 3**). In the same group, the genus *Actinomyces* positively associated with fecal calprotectin levels (q = 0.032).

## Discussion

Our study addresses associations of gut inflammation, microbial composition, and systemic inflammation in HIV-infected lactating women on cART in SSA where data have been scarce. None of the women in our study suffered from AIDS or HIV-related symptoms, and the levels of systemic inflammation were low. Therefore, no strong HIV-related alterations were observed; however, we would like to emphasize the following key results.

1. In our study population, similar levels of gut inflammation (fecal calprotectin levels), microbial translocation (LBP levels), and important descriptors of systemic inflammation (TNF, IL-6, IL-8, and CRP) were found in HIV-infected and HIV-uninfected individuals.
2. HIV infection can affect intestinal inflammation in more immune-compromised individuals, because fecal calprotectin levels correlated with third trimester CD4+ T-lymphocyte counts.
3. HIV-infected participants showed increased levels monocyte activation (increased sCD14 levels), despite taking cART and prophylactic antibiotics.
4. There is a relationship between intestinal inflammation, microbial translocation, and systemic inflammation because fecal calprotectin, LBP, and sCD14 correlated with biomarkers of systemic inflammation.
5. Stratified by HIV status, the gut microbiota differed in beta diversity but not alpha diversity.
6. Several gut microbial taxa were significantly associated with HIV status and systemic inflammation.

### Effect of HIV infection on biomarkers of gut inflammation and microbial translocation

In our population of asymptomatic HIV-infected and HIV-uninfected lactating women, intestinal inflammation (as assessed by fecal calprotectin levels) was not affected by HIV status. Subgroups with elevated fecal calprotectin levels included women with third-trimester CD4+ T-lymphocyte counts <350 cells/µL consistent with findings from previous studies (35, 36). Other studies in HIV-infected individuals described similar (37, 38) or elevated fecal calprotectin levels (8, 9), most likely depending on the degree of gut mucosal damage and immune dysfunction despite taking cART (39). In our study, above normal median fecal calprotectin levels were observed in HIV-infected cART-experienced individuals, in agreement with studies in cART-naïve Italians (40, 41).

In line with an overall well-preserved intestinal barrier with similar translocation of LPS and microbial antigens into the systemic circulation, we found no effects of HIV status on plasma LBP levels in our population. This contradicts previous studies showing elevated plasma LBP levels in HIV-infected adults from Europe and Africa (3, 10). However, our findings resembled results from studies in Ugandan, American, and Chinese adults (8, 42–44). Moreover, a study in Swedish HIV-infected adults revealed a decrease in LBP upon the commencement of cotrimoxazole prophylaxis (45). However, plasma LBP correlated positively with fecal calprotectin levels in some of our analyses, confirming that gut inflammation can impact the translocation of microbes and/or LPS in HIV-infected individuals on cART.

HIV-infected individuals in our study showed activation of innate immunity, indicated by persistently high plasma sCD14 levels, a biomarker for innate immune activation including monocytes and macrophages (46), and an independent predictor for mortality in HIV-infected individuals on cART (47, 48). Elevated sCD14 levels were also found in HIV-infected European and African children as well as African and Chinese adults (2, 3, 8, 13, 36, 43, 44, 46, 49, 50). We did not determine the source of monocyte activation in our population but our results are consistent with HIV-induced microbial translocation potentially driving monocyte/macrophage activation as demonstrated in another study (47).

In our study, plasma sCD14 levels did not differ significantly by antibiotic use and correlated neither with fecal calprotectin nor with plasma LBP levels. These findings are inconsistent with another study in cART-naïve Ugandan adults with recent HIV infection, which reported significant correlations between LBP and sCD14 levels (51). Differences between both study populations and/or cotrimoxazole prophylaxis in our study might explain this discrepancy; however, this warrants further investigations.

### Association of systemic inflammation biomarkers with HIV infection, microbial translocation, and gut inflammation

In line with an overall preserved immune function in our study population, we found overall similar plasma levels of important systemic inflammatory biomarkers (TNF, IL-6, IL-8, and CRP) in HIV-infected and HIV-uninfected women, consistent with previous work (2, 17, 52). Our results thus likely reflect protection from systemic inflammation due to cART treatment, as observed in other studies (14, 53). The situation was shown to be different in cART-naïve and more immune-suppressed cART-experienced individuals who showed increased levels of inflammatory biomarkers (49, 53–57).

However, our detailed analyses revealed some effects of HIV infection on the intestinal immune system: Fecal calprotectin positively correlated with proinflammatory cytokines in the HIV-infected group, consistent with findings from another study (8). Furthermore, in the HIV-infected group, LBP, a biomarker of microbial translocation, positively correlated with IL-2, IL-6, 1L-1β, IFN-γ, and CRP. Moreover, as shown in some (58, 59) but not all previous studies (60), CD4+ T-lymphocyte counts inversely correlated with LBP, CRP, and IL-6. These findings indicate some effects of gut inflammation and microbial translocation on systemic inflammation mainly in the more immune-compromised individuals in our study.

Furthermore, some cytokines seem to be sensitive to low-level HIV viremia; we observed a strong correlation of third-trimester HIV RNA levels with IL-4, IL-10, IL-6, IL-13, IL-1β, IL-13, IL-12p70, and TNF. Similar findings were also observed in suppressed and unsuppressed HIV-infected French and African participants (51, 61). Some effects of HIV infection on the immune system are likely mediated by macrophage/monocyte activation and inflammation (62). In our and a previous Spanish study (4), the monocyte/macrophage activation marker sCD14 correlated with IL-2, IL-6, TNF, and CRP.

Microbial translocation might also be relevant in the HIV-uninfected group, where we found a positive association of taxa from the Actinobacteriota phylum with LBP and fecal calprotectin, as well as the genus *Actinomyces* with fecal calprotectin in line with existing knowledge (63).

### The gut microbiota and its association with microbial translocation, gut, and systemic inflammation biomarkers

HIV infection can lead to a decrease in gut microbiota diversity, potentially resulting in the loss of beneficial bacteria and the proliferation of harmful ones (64). In contrast, the gut microbiota richness and evenness in our study were similar between the HIV-infected and HIV-uninfected groups in line with previous studies in cART-treated American and Spanish adults as well as South African and Italian children (37, 50, 65–68). In contrast, in other studies, reduced (16, 17, 64, 69) or increased (41) alpha diversity was observed in HIV-infected cART-naïve individuals. Furthermore, in our study, microbiota richness or evenness was not influenced by immune status, in agreement with some (34, 44, 70, 71) but not all previous studies in HIV-infected individuals (72). Most likely, cART-mediated viral suppression can restore or preserve immune status sufficiently that gross effects of HIV on the gut microbiota are no longer detectable.

We observed a decrease in microbial richness in the HIV-uninfected group from pregnancy to 6 weeks postpartum, possibly related to effects of birth and lactation. We also observed increasing richness in the HIV-infected group from pregnancy to 6 months after birth. The increase could have been an effect of better maternal compliance with cART in pregnancy and lactation to prevent HIV mother-to-child transmission, which would also drive a partial restoration of the gut-associated lymphoid tissue. However, in other studies, cART use caused substantial alterations to the composition of the gut microbiota (41) and a decrease in alpha diversity with time (73, 74).

We show significant inter-community differences (beta diversity) of the gut microbiota between HIV-uninfected and HIV-infected lactating women on cART. Similar findings were observed in cART-treated American, Spanish, and Chinese adults as well as South African and Italian cART-treated children (37, 44, 50, 64, 66, 67, 75). However, no significant variability in gut microbiota composition due to HIV was found in other studies (17, 68), possibly related to varying degrees of immune dysfunction or co-infection in the study populations.

At phylum level, we found high abundance of Firmicutes, Actinobacteriota, and Proteobacteria in all participants as previously reported in Asian and American cART-treated adults (44, 68, 76). Increased relative abundances of taxa from the Actinobacteriota phylum were observed in the HIV-infected group at 6 weeks postpartum in line with a Japanese study in cART-treated adults (76).

We found a higher relative abundance of the order Coriobacteriales and the genera *Collinsella* and *Slackia* in the HIV-infected group, consistent with previous findings in Zimbabwean children and cART-experienced Japanese participants (75, 76). The genus *Collinsella* has been previously linked to detrimental outcomes such as obesity, non-alcoholic steatohepatitis, and dyslipidemia (77). The lower relative abundance of genus *Collinsella* in the HIV-infected group noted by Zhao et al. was inconsistent with our results but concurred with other studies in American and Chinese adults (44, 68).

At genus level, we observed lower relative abundance of genus *Clostridium sensu_stricto_1* in the HIV-infected group at 6 weeks postpartum, consistent with previous studies in African and Chinese adults (44, 59)*. Clostridium* species are important obligate anaerobes in the human gut with a significant role in fermentation and metabolism of carbohydrates and amino acids. In another Chinese study, *Clostridium sensu_stricto_1* positively correlated with CD4+ and CD8+ T-lymphocytes counts, suggesting this taxon as a potential marker of improved immune status in HIV-infected participants (70).

In our study, the relative abundance of the genera *Romboutsia* and *Clostridium sensu_stricto_1* was lower in the HIV-infected group at 6 months postpartum in line with a study in South African children (37). The significantly lower relative abundances of *Romboutsia* and *Clostridium sensu_stricto_1* in the HIV-infected women may signify compromised metabolism and imbalanced intestinal homeostasis compared with the HIV-uninfected peers. In line with the beneficial role of *Clostridium* species, we found a negative association of the genus *Clostridia_UCG.014* with IFN-γ in the HIV-uninfected at 6 months postpartum, supporting a role in attenuating inflammation within the human gut.

More than 50% of the HIV-infected participants in our study were taking cotrimoxazole prophylaxis following the local and WHO guidelines (78). In our study, cotrimoxazole prophylaxis had no significant effects on general descriptors of the gut microbiota consistent with findings from a study in HIV-uninfected Ugandan adults and children (79, 80). These findings were confirmed with antibiotics other than cotrimoxazole in HIV-infected and HIV-uninfected individuals (69). However, in a study in African children, significant differences were noted in seven species of the gut microbiota with lower abundances in the cotrimoxazole treated group (80).

Assessing the relationship between HIV-associated microbial dysbiosis and systemic inflammation, we identified significant correlations between the relative abundance of certain taxa and plasma inflammatory biomarkers, warranting further investigations. Our findings were inconsistent with studies in Australian cART-naïve adults and Italian cART-experienced children where correlation of gut microbiota with systemic cytokines and microbial translocation markers was not found (17, 50).

### Strengths and limitations of the study

The strengths of our study lie in its comprehensive approach, encompassing longitudinal assessments of multiple intestinal and systemic inflammatory biomarkers, as well as the gut microbiota in HIV-infected and HIV-uninfected controls from the same community. However, there are limitations that should be acknowledged. First, because of the inclusion criteria of our study, we were unable to disentangle the effects of cART and HIV infection, as all HIV-infected women were receiving cART. Furthermore, the relatively small sample size hampers the generalizability of our conclusions, and it is possible that certain associations between microbial taxa and inflammatory biomarkers may have been overlooked. We are therefore also underpowered to draw meaningful conclusions from cART duration and HIV viremia group comparisons. Moreover, our study exclusively comprised lactating female participants, with no age-matched non-lactating controls that may restrict the generalizability of our findings to other populations. In addition, we did not directly assess microbial translocation, and measurements of 16S rDNA levels, as previously done (15) and could be used in follow-up studies. Finally, non-bacterial infections, such as protozoa, were not tested in our study.

## Conclusion

In our study population of HIV-infected lactating women on cART without AIDS and HIV-related symptoms, many important parameters of the immune system were not affected by HIV. However, we identified effects of HIV on gut inflammation, microbial composition, and translocation as well as some cytokines, in line with a role of intestinal pathology contributing to systemic inflammation in HIV.

## Data availability statement

The data presented in this manuscript are tabulated in the main paper and **Supplementary Materials**. All sequencing data generated in the preparation of this manuscript, files and entire details of used samples used has been deposited in https://doi.org/10.6084/m9.figshare.24455392.v1.

## Ethics statement

The studies involving humans were approved by The Joint Parirenyatwa Hospitals and University of Zimbabwe Research Ethics Committee (JREC), JREC/114/20 and the Medical Research Council of Zimbabwe (MRCZ), MRCZ/A/2663. The studies were conducted in accordance with the local legislation and institutional requirements. The participants provided their written informed consent to participate in this study.

## Author contributions

PM: Conceptualization, Formal Analysis, Investigation, Methodology, Writing – original draft. PC: Investigation, Methodology, Writing – review & editing. JW: Formal Analysis, Writing – review & editing. AM: Methodology, Writing – review & editing. SJ: Formal Analysis, Writing – review & editing. RG: Writing – review & editing. LK: Conceptualization, Writing – review & editing. BY: Funding acquisition, Methodology, Supervision, Writing – review & editing. BM: Conceptualization, Funding acquisition, Investigation, Supervision, Writing – review & editing. KD: Conceptualization, Investigation, Supervision, Writing – review & editing.

## Supplementary material

Supplementary Figure 1 Plasma proinflammatory and vascular injury biomarkers in HIV infected and HIV uninfected women. Comparison of plasma proinflammatory and vascular injury biomarkers in HIV-infected vs. HIV uninfected women at 6 weeks postpartum and 6 months postpartum. Significance after Bonferroni correction is indicated as non-significant (ns), q<0.05 (*), q< 0.01 (**) or q< 0.001 (***). CRP, c-reactive protein; ICAM, intercellular adhesion molecule; IL, Interleukin; TNF, tumour necrosis factor; INF-γ, interferon gamma; VCAM, vascular cell adhesion molecule.

Supplementary Figure 2 Comparison of pregnancy, 6 weeks and 6 months postpartum gut microbiota. Alpha diversity comparison in HIV-uninfected women **(A)** and HIV-infected women **(B)**. Beta diversity comparison in HIV-uninfected women **(C)** and HIV-infected women **(D)**.
