# Supplementary figures and images for: Immune activation and inflammation in lactating women on combination antiretroviral therapy: role of gut dysfunction and gut microbiota imbalance
Source: Front Immunol. 2023 Nov 16;14:1280262. doi: 10.3389/fimmu.2023.1280262 (PMC10693333; doi:10.3389/fimmu.2023.1280262)

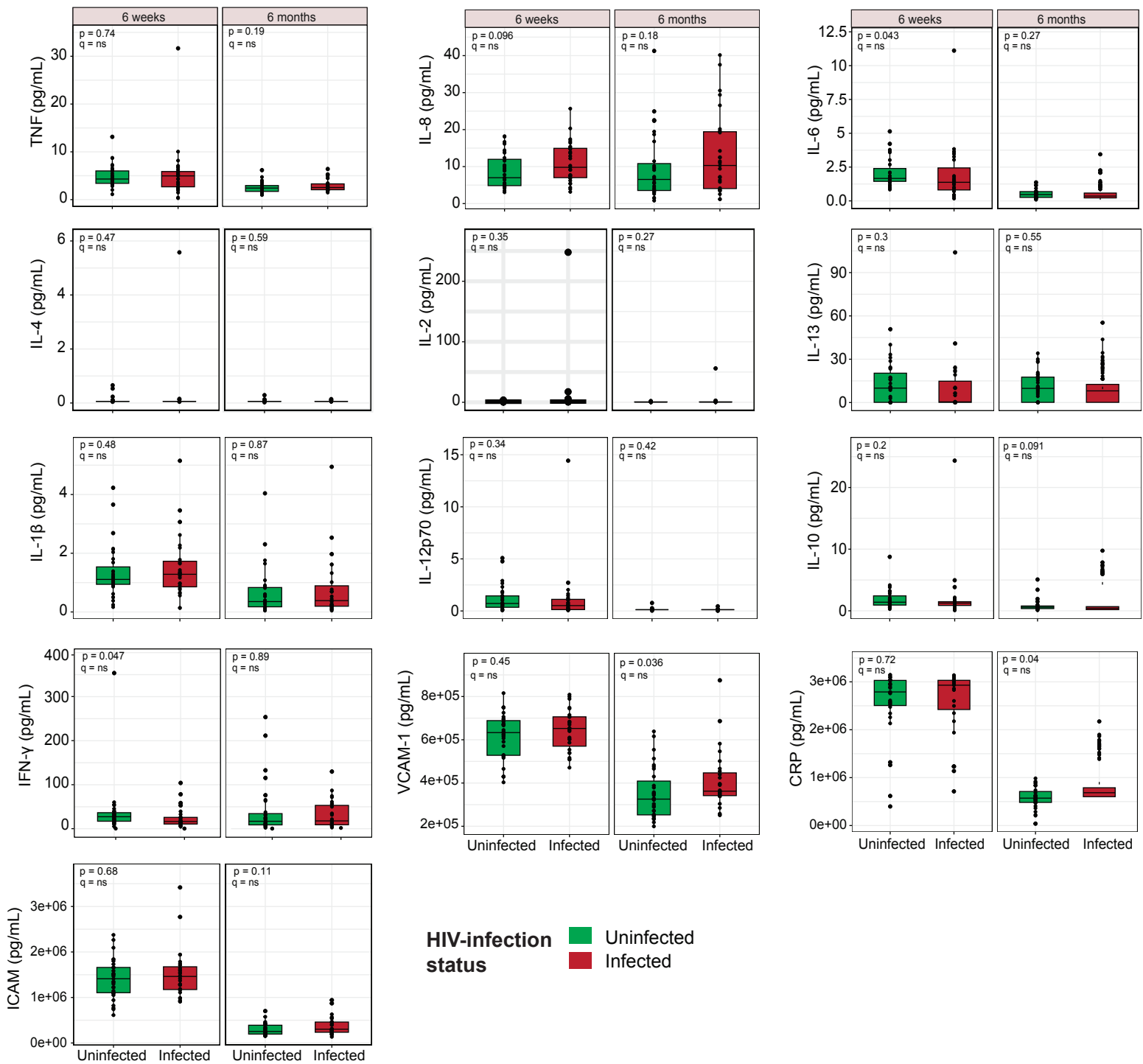

Supplement: Supplementary Figure 1 — Plasma proinflammatory and vascular injury biomarkers in HIV infected and HIV uninfected women. Comparison of plasma proinflammatory and vascular injury biomarkers in HIV-infected vs. HIV uninfected women at 6 weeks postpartum and 6 months postpartum. Significance after Bonferroni correction is indicated as non-significant (ns), q<0.05 (*), q< 0.01 (**) or q< 0.001 (***). CRP, c-reactive protein; ICAM, intercellular adhesion molecule; IL, Interleukin; TNF, tumour necrosis factor; INF-γ, interferon gamma; VCAM, vascular cell adhesion molecule. [file DataSheet_1.pdf]

**A**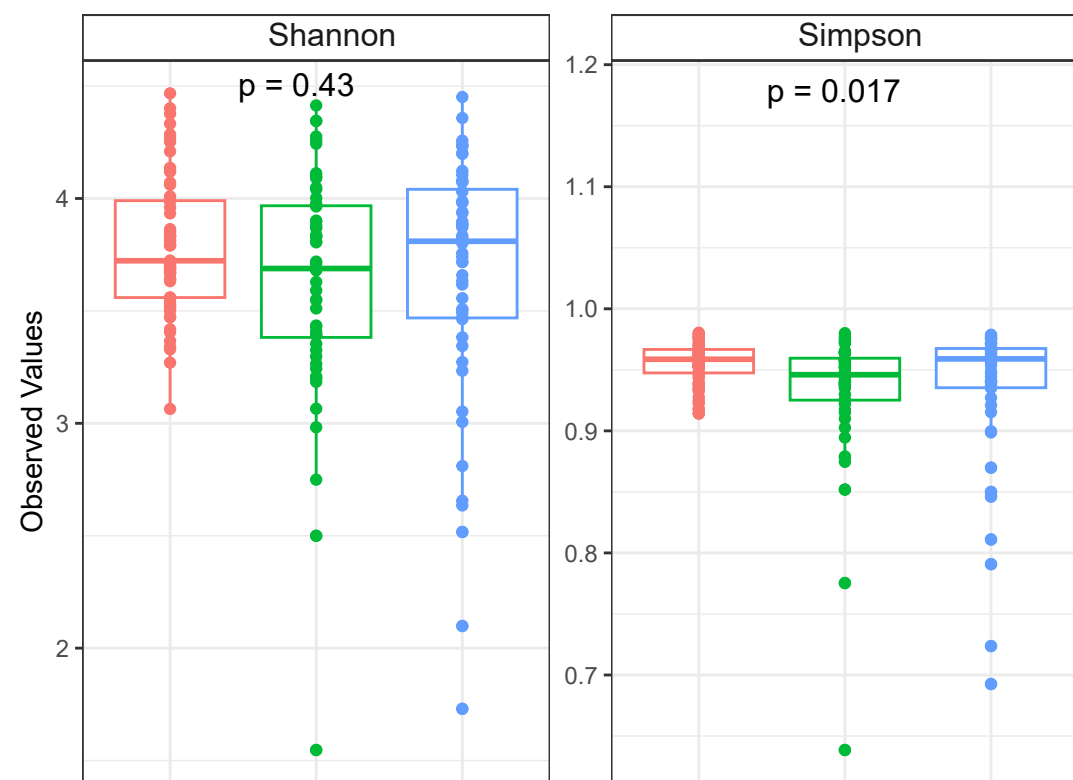**B**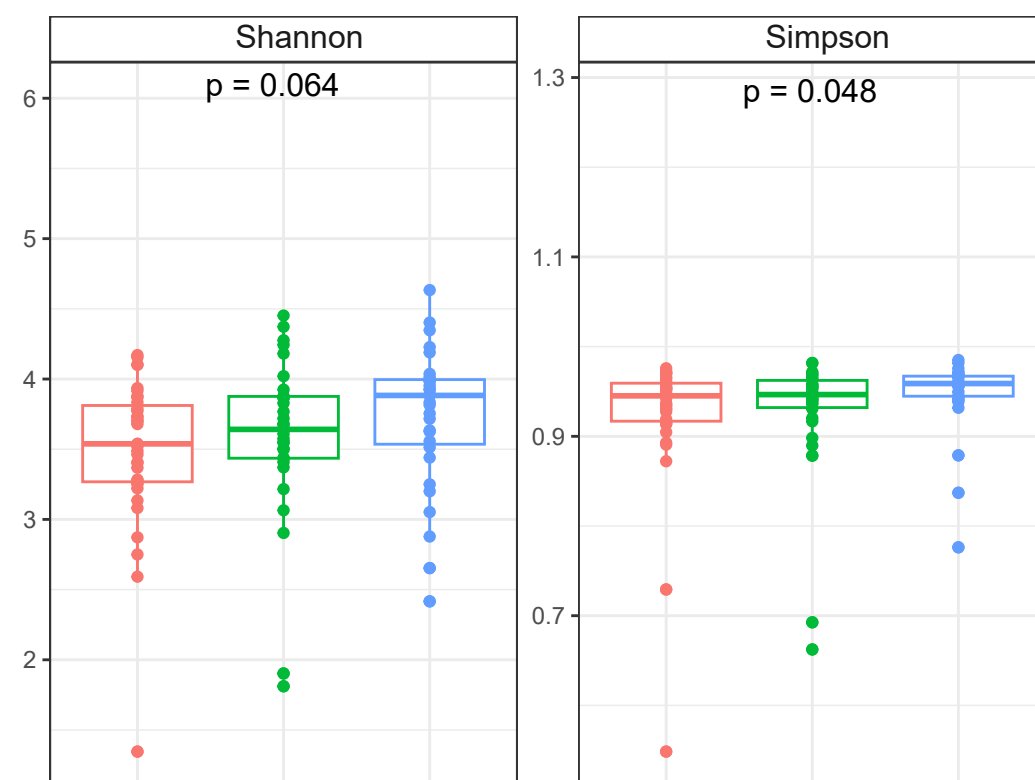**C**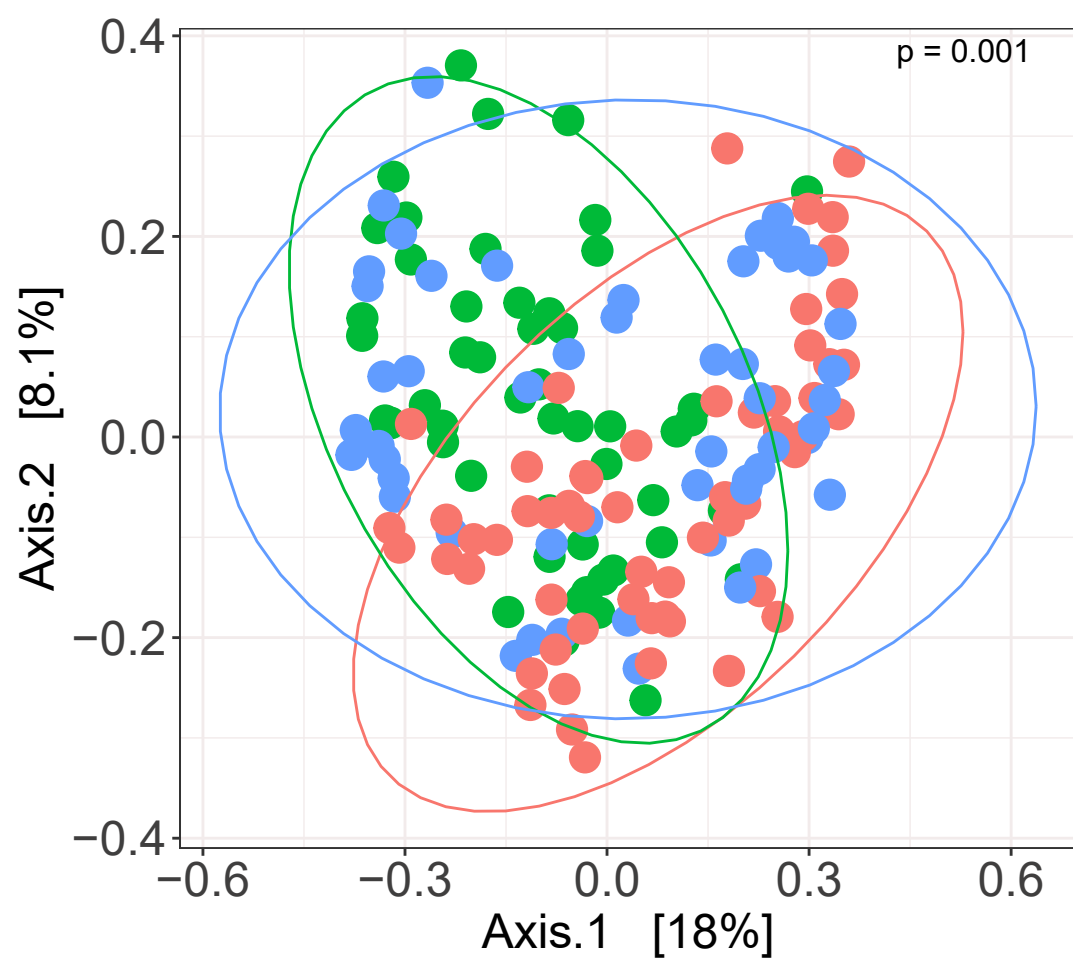**D**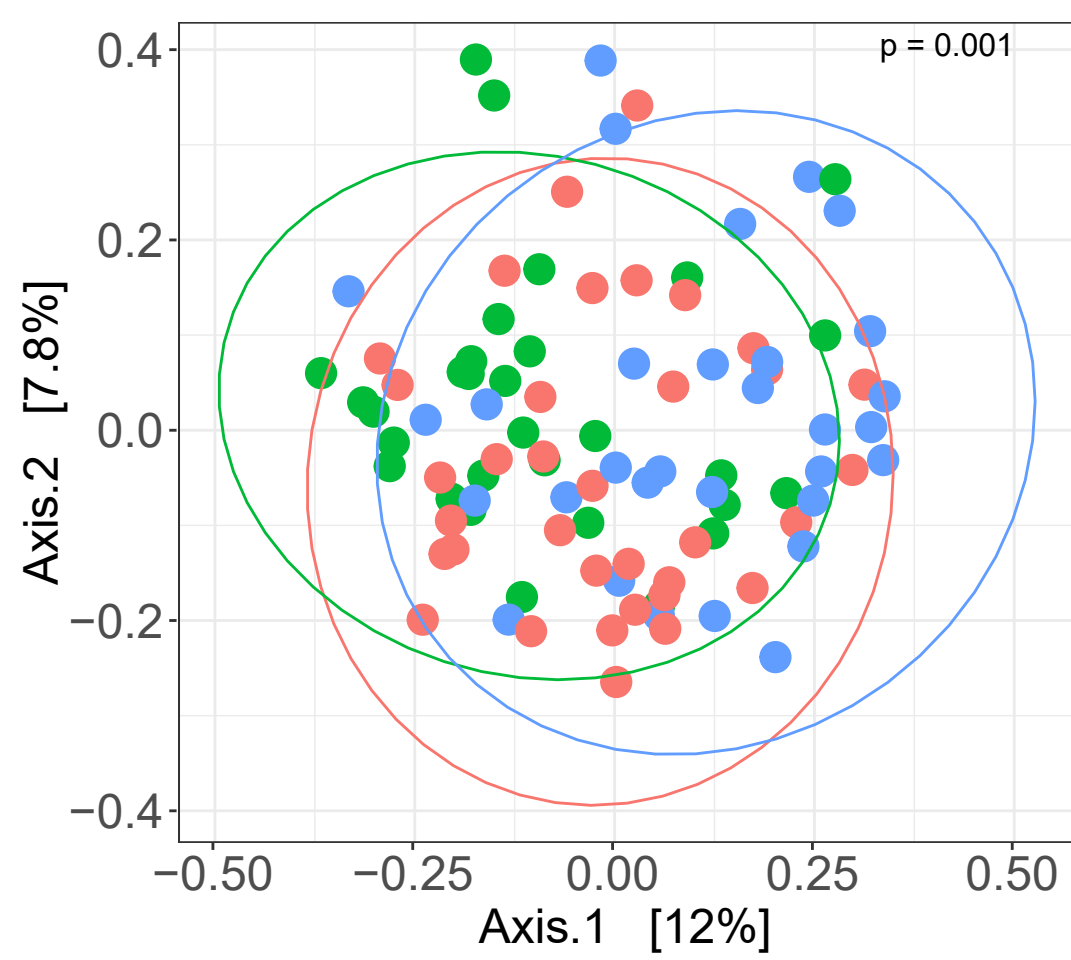

Visit

- Pregnancy
- 6 weeks postpartum
- 6 months postpartum

Supplement: Supplementary Figure 2 — Comparison of pregnancy, 6 weeks and 6 months postpartum gut microbiota. Alpha diversity comparison in HIV-uninfected women (A) and HIV-infected women (B). Beta diversity comparison in HIV-uninfected women (C) and HIV-infected women (D). [file DataSheet_2.pdf]
